# Supplementary material for: Development and Identification of Novel α-Glucosidase Inhibitory Peptides from Mulberry Leaves
Source: Foods. 2023 Oct 26;12(21):3917. doi: 10.3390/foods12213917 (PMC10649714; doi:10.3390/foods12213917)
Supplement: Supplementary file 1 [file foods-12-03917-s001.zip › foods-2647336-supplementary.pdf]

Supplementary information

Table S1. Parameters levels in optimization.

Parameters	Levels		
	**-1**	**0**	**1**
**Temperature / ˚C (A)**	45	55	65
**Hydrolysis Time / h (B)**	3	4.5	6
**Enzyme Amount / U/g (C)**	10000	15000	20000

Table S2. Response surface method (RSM) design and results.

Std	Run	A: Temperature (°C)	B: Time (h)	C: Enzyme Amount (U/g)	DH (%)
16	1	55	4.5	15000	26.76
4	2	65	6	15000	22.01
3	3	45	6	15000	24.96
13	4	55	4.5	15000	26.29
17	5	55	4.5	15000	26.52
12	6	55	6	20000	26.12
14	7	55	4.5	15000	26.33
2	8	65	3	15000	21.41
1	9	45	3	15000	21.05
8	10	65	4.5	20000	22.57
6	11	65	4.5	10000	20.56
9	12	55	3	10000	22.04
15	13	55	4.5	15000	26.92
7	14	45	4.5	20000	24.57
10	15	55	6	10000	24.28
5	16	45	4.5	10000	22.88
11	17	55	3	20000	24.47

Table S3. Identified results of fraction 1 by LC-MS/MS.

No.	Sequence	Length	Charge	m/z	Mass
1	VVLYKSLQ	8	2	475.29	948.56
2	KITFPILF	8	2	489.80	977.59
3	RRRSLRKK	8	3	367.25	1098.72
4	VRPRGGPG	8	2	398.23	794.45
5	AAGRLPGY	8	2	402.72	803.43
6	TVKPAYFGV	9	2	491.27	980.53
7	RIIKNKKK	8	2	514.36	1026.70
8	SDRIPPIIGL	10	2	540.82	1079.63
9	KSLKGVGR	8	2	422.77	843.53
10	LTGPIPEF	8	2	437.24	872.46
11	ISILAELV	8	2	429.27	856.53
12	VLPAHKFG	8	2	434.76	867.50
13	VFKVRVTV	8	2	474.31	946.60
14	IQYYFLRP	8	2	550.30	1098.59
15	SEGLVLAG	8	1	745.41	744.40
16	LPSRALHI	8	2	453.78	905.54
17	PLDDNVIP	8	2	441.73	881.45
18	LQFPDEYLVK	10	2	626.33	1250.65
19	VLTVVLLV	8	2	428.30	854.58
20	IVSARCAA	8	2	395.72	789.42
21	TASKLLLR	8	2	451.30	900.58
22	VGQSEKKK	8	2	452.27	902.52
23	YVIRKRIK	8	3	359.24	1074.70
24	LAWVVKSH	8	2	470.27	938.53
25	LASVNGHK	8	2	413.23	824.45
26	ITYTILIE	8	2	483.28	964.55
27	TIYTVFLP	8	2	477.27	952.53

28	PKGRTGKV	8	2	421.76	841.51
29	HRRHTGVL	8	2	488.28	974.55
30	LDVLLVLP	8	2	441.29	880.56
31	VVRDFHNA	8	2	479.25	956.48
32	VAIAITIP	8	2	399.26	796.51
33	WVILLTTA	8	2	458.78	915.54
34	HGALGFLR	8	2	435.75	869.49
35	VKHRYRIA	8	2	521.82	1041.62
36	VTRPQLRR	8	2	513.32	1024.63
37	RRPQREVV	8	2	520.31	1038.60
38	LFYRRARK	8	2	555.34	1108.66
39	VFPKQHIF	8	2	508.29	1014.57
40	VGINCAPP	8	2	385.70	769.38
41	SSFDDALL	8	1	867.41	866.40
42	AYYYNADQ	8	2	504.21	1006.40
43	EEEDKKVE	8	2	503.24	1004.47
44	DDLYARQH	8	2	509.24	1016.47
45	LVLRGRVF	8	2	480.31	958.61
46	PPVLPLLT	8	2	425.28	848.54
47	WPILIKHL	8	2	510.32	1018.63
48	KVLKHQKG	8	2	469.30	936.59
49	RWPFFAFM	8	2	551.27	1100.53
50	SALPVGIW	8	2	421.74	841.47
51	LFVVLYVP	8	2	475.29	948.57
52	WQRGRRHH	8	3	378.20	1131.59
53	QVDKVLLP	8	2	456.28	910.55
54	KKYWILFR	8	3	385.23	1152.68
55	VVKDHGSF	8	2	444.73	887.45
56	RRYVRQLP	8	2	544.33	1086.64

57	EGSIDFIFG	9	1	984.47	983.46
58	VLKGHKLA	8	2	433.28	864.55
59	PFGSPTAV	8	2	388.20	774.39
60	PFKKLWIL	8	2	522.83	1043.65
61	MTWQILFR	8	2	547.79	1093.57
62	YKYTKNIIIE	10	2	642.86	1283.71
63	SFRKMKKL	8	2	519.32	1036.62
64	SLPIENGLIV	10	1	1054.61	1053.61
65	SDVYAPRS	8	2	447.72	893.42

Table S4. Distribution of free binding energy levels for molecular docking (unit: kcal/mol).

ranking / peptides	1	2	3	4	5	6	7	8	9	10
LFYRRARK	-8.81	-8.35	-8.34	-8.32	-8.21	-8.17	-8.14	-8.00	-7.93	-7.91
AAGRLPGY	-8.79	-8.72	-8.69	-8.38	-8.15	-8.13	-8.10	-8.04	-8.04	-8.03
VVRDFHNA	-8.78	-8.55	-8.53	-8.50	-8.44	-8.36	-8.34	-8.30	-8.30	-8.29
RWPFFAFM	-8.75	-8.71	-8.71	-8.69	-8.68	-8.65	-8.64	-8.59	-8.56	-8.51
VGINCAPP	-8.68	-8.58	-8.40	-8.17	-8.16	-8.15	-8.13	-8.13	-8.04	-8.03
SALPVGIW	-8.62	-8.49	-8.37	-8.30	-8.29	-8.28	-8.26	-8.15	-8.13	-8.11
VFPKQHIF	-8.58	-8.17	-8.16	-8.15	-8.15	-8.10	-8.06	-8.04	-8.02	-7.92
SDVYAPRS	-8.54	-8.47	-8.25	-8.06	-8.04	-8.03	-8.01	-7.91	-7.90	-7.89
RRYVRQLP	-8.53	-8.40	-8.29	-8.27	-8.24	-8.22	-8.18	-8.13	-8.04	-8.04
VLPAHKFG	-8.43	-8.33	-8.27	-8.26	-8.24	-8.23	-8.13	-8.12	-8.10	-8.00
PFGSPTAV	-8.27	-8.14	-8.04	-7.86	-7.83	-7.79	-7.70	-7.60	-7.57	-7.53
VRPRGGPG	-8.27	-8.18	-8.08	-8.08	-8.01	-8.00	-7.99	-7.99	-7.97	-7.93
VTRPQLRR	-8.13	-7.92	-7.91	-7.87	-7.84	-7.81	-7.59	-7.57	-7.54	-7.38
PKGRTGKV	-8.11	-7.84	-7.48	-7.42	-7.41	-7.40	-7.37	-7.36	-7.35	-7.32
TIYTVFLP	-8.10	-8.09	-7.88	-7.84	-7.78	-7.77	-7.72	-7.69	-7.69	-7.64
WQRGRRHH	-8.02	-7.99	-7.94	-7.93	-7.92	-7.90	-7.86	-7.86	-7.81	-7.73
PLDDNVIP	-8.02	-7.98	-7.89	-7.88	-7.82	-7.81	-7.73	-7.69	-7.64	-7.50
MTWQILFR	-8.01	-7.91	-7.81	-7.77	-7.70	-7.66	-7.64	-7.62	-7.57	-7.57
WVILLTTA	-7.99	-7.99	-7.97	-7.97	-7.91	-7.87	-7.85	-7.81	-7.81	-7.74
VKHRYRIA	-7.96	-7.69	-7.60	-7.56	-7.55	-7.54	-7.47	-7.47	-7.44	-7.34
VLTVVLLV	-7.91	-7.70	-7.69	-7.65	-7.52	-7.49	-7.40	-7.34	-7.31	-7.22
RRPQREVV	-7.87	-7.73	-7.72	-7.71	-7.62	-7.58	-7.53	-7.49	-7.49	-7.44
SLPIENGLIV	-7.85	-7.83	-7.65	-7.45	-7.43	-7.40	-7.37	-7.36	-7.25	-7.25
YVIRKRIK	-7.84	-7.66	-7.56	-7.54	-7.46	-7.46	-7.44	-7.43	-7.43	-7.40
ISILAELV	-7.84	-7.72	-7.71	-7.68	-7.65	-7.58	-7.55	-7.54	-7.50	-7.45
IQYYFLRP	-7.84	-7.77	-7.74	-7.64	-7.62	-7.59	-7.56	-7.55	-7.54	-7.52

**AYYYNADQ**	-7.79	-7.79	-7.68	-7.68	-7.62	-7.57	-7.56	-7.48	-7.48	-7.45
**LTGPIPEF**	-7.79	-7.78	-7.65	-7.62	-7.57	-7.54	-7.54	-7.50	-7.44	-7.42
**HGALGFLR**	-7.77	-7.68	-7.60	-7.58	-7.54	-7.54	-7.49	-7.47	-7.42	-7.34
**DDLYARQH**	-7.77	-7.70	-7.64	-7.64	-7.61	-7.39	-7.39	-7.38	-7.38	-7.29
**YKYTKNIIIE**	-7.73	-7.46	-7.45	-7.43	-7.42	-7.41	-7.39	-7.38	-7.37	-7.37
**KKYWILFR**	-7.73	-7.68	-7.57	-7.56	-7.56	-7.51	-7.49	-7.42	-7.35	-7.34
**LVLRGRVF**	-7.72	-7.50	-7.43	-7.42	-7.39	-7.33	-7.33	-7.30	-7.29	-7.27
**ITYTILIE**	-7.72	-7.67	-7.43	-7.42	-7.40	-7.37	-7.34	-7.30	-7.25	-7.25
**LPSRALHI**	-7.67	-7.40	-7.40	-7.38	-7.36	-7.36	-7.32	-7.31	-7.29	-7.28
**TVKPAYFGV**	-7.67	-7.52	-7.24	-6.97	-6.97	-6.94	-6.87	-6.86	-6.84	-6.83
**PPVLPLLT**	-7.64	-7.59	-7.51	-7.42	-7.38	-7.36	-7.31	-7.28	-7.22	-7.16
**SEGLVLAG**	-7.58	-7.43	-7.40	-7.37	-7.37	-7.36	-7.34	-7.33	-7.30	-7.24
**RRRSLRKK**	-7.57	-7.44	-7.33	-7.30	-7.28	-7.28	-7.26	-7.15	-7.14	-7.09
**QVDKVLLP**	-7.54	-7.32	-7.26	-7.15	-7.12	-7.05	-7.02	-7.01	-6.93	-6.91
**IVSARCAA**	-7.51	-7.41	-7.34	-7.27	-7.26	-7.24	-7.16	-7.16	-7.16	-7.09
**KVLKHQKG**	-7.50	-7.43	-7.40	-7.39	-7.39	-7.37	-7.35	-7.33	-7.29	-7.29
**PFKKLWIL**	-7.50	-7.42	-7.36	-7.17	-7.11	-7.10	-7.01	-7.01	-7.00	-6.93
**VAIAITIP**	-7.48	-7.28	-7.25	-7.22	-7.10	-7.09	-7.03	-6.99	-6.95	-6.91
**SDRIPPIIGL**	-7.47	-7.06	-7.04	-7.04	-7.03	-7.00	-7.00	-6.94	-6.93	-6.93
**SSFDDALL**	-7.46	-7.24	-7.20	-7.19	-7.03	-6.96	-6.96	-6.94	-6.91	-6.86
**VFKVRVTV**	-7.37	-7.36	-7.29	-7.19	-7.09	-7.06	-6.96	-6.88	-6.80	-6.77
**LASVNGHK**	-7.33	-6.93	-6.84	-6.80	-6.65	-6.64	-6.62	-6.61	-6.60	-6.52
**VLKGHKLA**	-7.28	-7.28	-7.25	-7.24	-7.19	-7.15	-7.03	-6.97	-6.91	-6.87
**LDVLLVLP**	-7.24	-7.11	-7.01	-7.00	-6.99	-6.89	-6.86	-6.79	-6.69	-6.64
**LAWVVKSH**	-7.18	-7.17	-7.16	-6.94	-6.90	-6.87	-6.85	-6.83	-6.77	-6.76
**EGSIDFIFG**	-7.10	-7.05	-6.96	-6.96	-6.89	-6.85	-6.84	-6.76	-6.73	-6.71
**VVLYKSLQ**	-7.08	-7.03	-6.93	-6.75	-6.66	-6.65	-6.58	-6.53	-6.52	-6.42
**LQFPDEYLVK**	-7.07	-6.73	-6.65	-6.59	-6.57	-6.56	-6.54	-6.37	-6.33	-6.29
**KITFPILF**	-7.06	-7.04	-6.92	-6.91	-6.84	-6.83	-6.59	-6.53	-6.51	-6.50

**HRRHTGVL**	-7.06	-7.03	-7.03	-7.02	-7.01	-7.01	-7.01	-7.00	-6.98	-6.92
**VVKDHGSF**	-7.02	-6.94	-6.86	-6.86	-6.85	-6.85	-6.83	-6.78	-6.78	-6.67
**SFRKMKKL**	-7.00	-6.93	-6.68	-6.67	-6.56	-6.53	-6.49	-6.49	-6.42	-6.42
**VGQSEKKK**	-6.94	-6.85	-6.81	-6.81	-6.80	-6.78	-6.74	-6.70	-6.66	-6.57
**LFVVLYVP**	-6.93	-6.80	-6.80	-6.78	-6.73	-6.72	-6.65	-6.63	-6.61	-6.42
**RIIKNKKK**	-6.84	-6.70	-6.51	-6.48	-6.43	-6.42	-6.41	-6.40	-6.40	-6.34
**WPILIKHL**	-6.71	-6.63	-6.55	-6.52	-6.51	-6.44	-6.42	-6.42	-6.39	-6.35
**KSLKGVGR**	-6.71	-6.36	-6.31	-6.28	-6.24	-6.23	-6.20	-6.16	-6.13	-6.04
**TASKLLLR**	-6.26	-6.15	-6.12	-6.06	-6.05	-6.04	-6.03	-6.01	-5.99	-5.94
**EEEDKKVE**	-6.10	-6.09	-6.03	-6.00	-5.98	-5.92	-5.88	-5.87	-5.81	-5.77
**Acarbose**	-9.91	-9.26	-9.00	-8.80	-8.80	-8.80	-8.80	-8.60	-8.60	-8.40

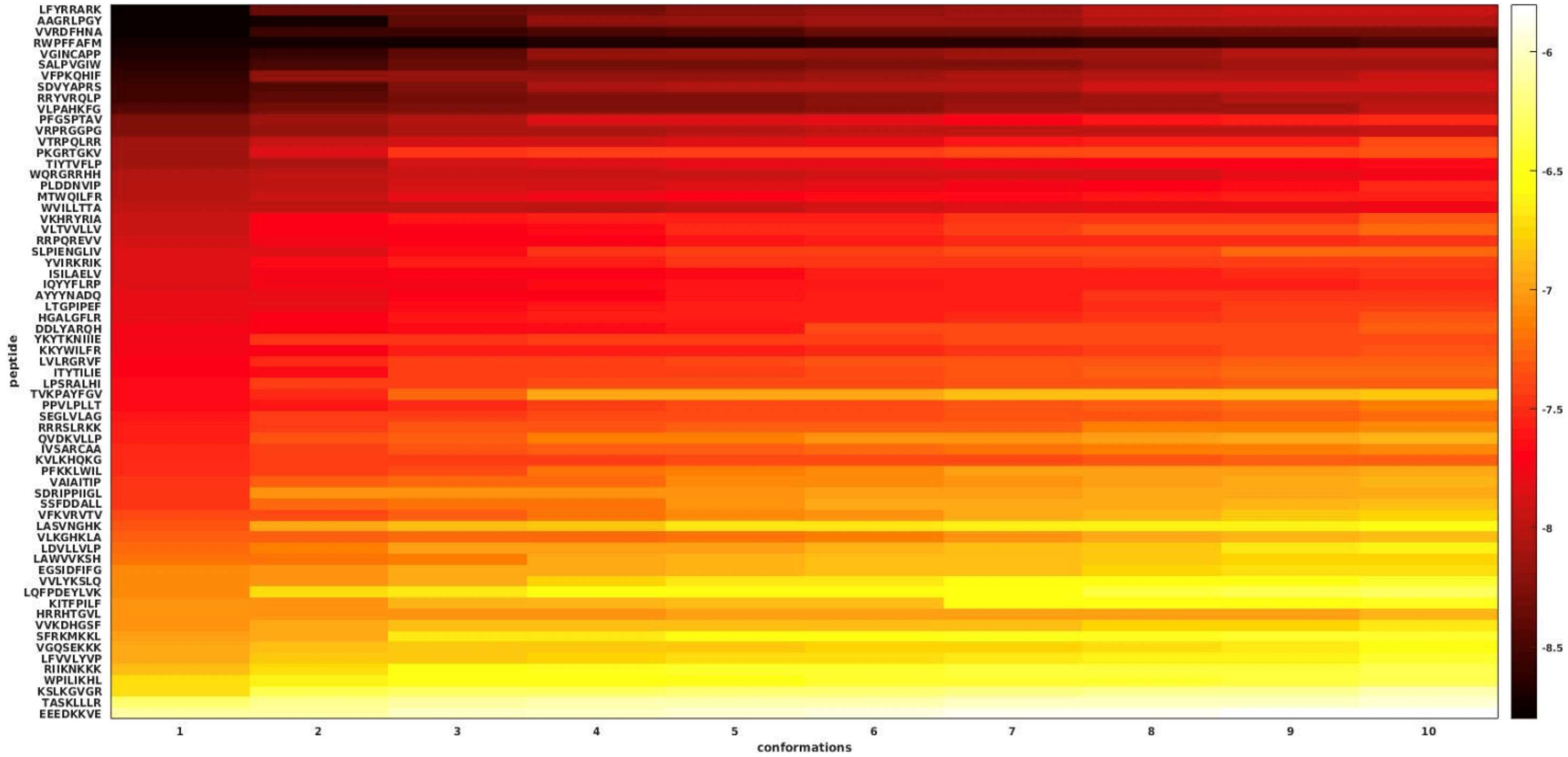

Figure S1. Heatmap of peptides-α-glucosidase binding free energy statistics. The darker color means the lower binding energy.
